# Polyparasitism with *Schistosoma haematobium, Plasmodium* and soil-transmitted helminths in school-aged children in Muyuka–Cameroon following implementation of control measures: a cross sectional study

**DOI:** 10.1186/s40249-021-00802-x

**Published:** 2021-02-17

**Authors:** Irene Ule Ngole Sumbele, Ofon Vitalis Otia, Orelien Sylvain Mtopi Bopda, Calvin Bisong Ebai, Helen KuoKuo Kimbi, Theresa Nkuo-Akenji

**Affiliations:** 1 Department of Zoology and Animal Physiology, University of Buea, Buea, Cameroon; 2 Department of Medical Laboratory Sciences, University of Buea, Buea, Cameroon; 3 Department of Medical Laboratory Science, University of Bamenda, Bambili, Cameroon; 4 Department of Microbiology and Parasitology, University of Buea, Buea, Cameroon; 5 Department of Microbiology and Immunology, College of Veterinary Medicine, Cornell University, Ithaca, NY USA

**Keywords:** Polyparasitism, School-age children, *Schistosoma haematobium*, *Plasmodium*, Soil-transmitted helminths, Morbidity, Risk factor, Cameroon

## Abstract

**Background:**

Despite the ubiquity of polyparasitism, its health impacts have been inadequately studied. The aim of this study was to determine the prevalence and determinants of polyparasitism with *Schistosom*a *haematobium*, *Plasmodium* and soil-transmitted helminths (STH) following sustained control measures, as well as evaluate the outcomes and clinical correlates of infection in school-aged children (SAC) living in the schistosomiasis endemic focus of Muyuka-Cameroon.

**Methods:**

In a cross-sectional study, urine, blood and stool samples were each collected from SAC (4–14 years) selected at random between March and June 2015. Microhaematuria in urine was detected using reagent strip and *S. haematobium* ova by filtration/microscopy methods. *Plasmodium* was detected using Giemsa-stained blood films and complete blood count was obtained using an auto-haematology analyser. STH in stool was detected by the Kato-Katz method. Categorical and continuous variables were compared as required, Kappa value estimated and the adjusted odds ratio (a*OR*) in the multivariate analysis was used to evaluate association of the risk factors with infection.

**Results:**

Out of the 638 SAC examined, single infection was prevalent in 33.4% while polyparasitism was 19.9%. Prevalence of *S. haematobium* + *Plasmodium* was 7.8%; *S. haematobium* + STH was 0.8%; *Plasmodium* + STH was 0.8%; while *S. haematobium* + *Plasmodium* + STH was 0.9%. Higher preponderance of *S. haematobium* + *Plasmodium* infection occurred in females, those from Likoko, did not use potable water, practiced bathing in stream and carried out open defecation than their equivalents. However, being female (a*OR* = 2.38, *P* = 0.009) was the only significant risk factor identified. Anaemia was a common morbidity (74.3%) with a slight agreement with microscopy in predicting *S. haematobium* and *Plasmodium* infections. The sensitivity and specificity of haematuria (13.0%) in predicting *S. haematobium* infection was 46.5% and 100% with a moderate agreement with microscopy. Co-infection with *S. haematobium* and malaria parasite was significantly associated with threefold odds of history of fever in the last three days.

**Conclusions:**

Polyparasitism is a public health problem in Muyuka with females most at risk. Anaemia prevalence is exacerbated in co- and triple-infections and together with a history of fever are of value in predicting polyparasitism.
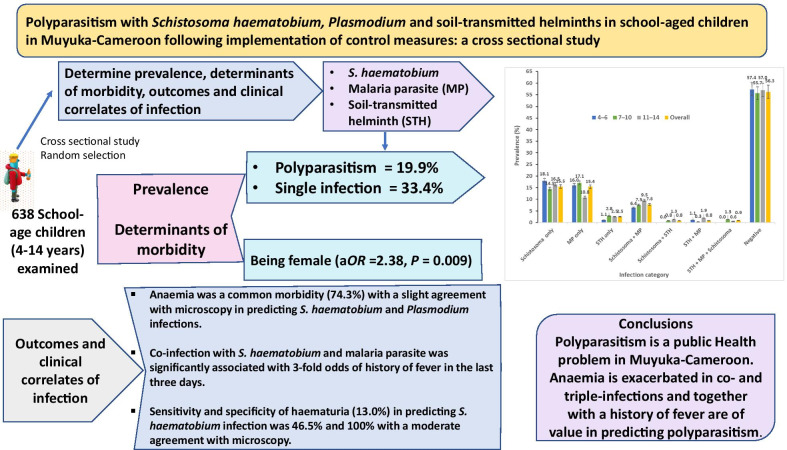

## Background

Polyparasitism is a common condition in human populations in which a person experiences disease from two or more concomitant, chronic infections with helminthic and/or protozoan parasites [1, 2]. In addition to malaria, schistosomiasis and soil-transmitted helminths (STH) constitute a major public health problem in many parts of sub-Saharan Africa. A total of 229.2 million people in 2018 needed preventive chemotherapy (PC) for schistosomiasis globally, of which 124.4 million were school-aged children [3]. More than 1.5 billion people are infected with STH worldwide and over 568 million school-aged children (SAC) live in areas of intensive transmission and need treatment and preventive interventions [4]. On the other hand, out of the 228 million malaria cases reported in 2018, 213 million (93%) occurred in the World Health Organisation (WHO) African Region [5].

As helminth and *Plasmodium* infections overlap geographically in developing countries, it is therefore a probable phenomenon for polyparasitism to occur causing high morbidity, however, these infections are rarely studied together. Neglected tropical diseases (NTD) such as schistosomiasis, a water-borne parasitic disease remains a focal disease while infection with STHs such as *Ascaris lumbricoides, Trichuris trichiura* and hookworms are ubiquitous in developing regions of Africa, Asia and the Americas [6]. In the ecological settings of Mount Cameroon, the transmission of *Schistosoma haematobium*, *Plasmodium* spp. and STH is common, and concurrent urogenital schistosomiasis, malaria, and/or ascariasis have been reported [7–9]. Universal factors attributed to the co-occurrence of these infections include poor sanitation, inadequate toilet facilities, lack of potable water, and ineffective public health enlightenment programme and services [8, 10].

Despite the ubiquity of polyparasitism, its health impacts have been inadequately studied. The effects of polyparasitism are often clinically inapparent, however concomitant infection of two parasites may modulate the effects of each other within their host [11]. While it is unclear if co-infections with helminths such as schistosomes can modulate susceptibility to malaria in humans [12], it is difficult to attribute morbidity-related outcomes in situations where multiple causative pathogens co-exist within the same person. Sparse evidence on the effect of polyparasitism in SAC is suggestive of the occurrence of anaemia, malnutrition, impaired cognitive development, splenomegaly, fatigue and multiplicative impact on organ pathology [2, 13–16]. These subtle morbidities like malnutrition as well as its common presentation stunting, anaemia, and leucocytosis are correlates of both helminths and protozoan infections [8, 17].

The control of schistosomiasis and STH in endemic regions in Africa rely on regular mass drug administration (MDA) and monitoring of infections in SAC. In 2015, 53.2 million and 417 million SAC received PC for schistosomiasis and STH, respectively [18]. With an estimated two million people with schistosomiasis in Cameroon and an additional five million living in high transmission areas, annual MDA of praziquantel to SAC in endemic areas is the country’s main control strategy against the disease [19, 20]. Among the integrated malaria control strategies in different epidemiological settings in Cameroon, vector control intervention through distribution of long-lasting insecticidal nets (LLINs) has been scaled-up [21]. While the prevalence of these parasites have experienced a reduction due to mapping of schistosomiasis endemic areas and sustained MDA campaigns with praziquantel, PC with albendazole for STH and the scale-up of treated bed nets across the country between 2000 and 2015 for control of malaria [20, 22, 23], these have not been sufficient in interrupting the transmission cycle of the parasites. Hence there is the need for regular monitoring studies in endemic areas to tailor strategies to ensure site-specific interruption of the disease transmission.

Due to the commonality of *Plasmodium* and helminth co-infections [24], improved understanding of polyparasitism is of concern in resource-limited settings in developing countries where diagnosis and treatment strategies are prioritized. Hence, investigating the implications of morbidities associated with polyparasitism is invaluable for healthcare workers. The aim of this study was to determine the prevalence and determinants of polyparasitism with *S. haematobium*, *Plasmodium* and STH following sustained control measures as well as evaluate the outcomes and the clinical correlates of infection in SAC living in the schistosomiasis endemic foci of Bafia, Ikata and Likoko in Muyuka, Cameroon.

## Methods

### Study area and participants

This study was carried out in three rural urogenital schistosomiasis endemic localities of Ikata, Bafia and Mile 14-Lykoko in the Muyuka Health District, Cameroon. A detailed description of the study sites has been reported previously [25]. While there is potable water and streams in Bafia and Ikata, Likoko Native has no potable water. The villages have an integrated health centre (IHC) each except for Likoko. In addition to the IHC, Bafia has another health centre belonging to the Cameroon Baptist Convention. Previous studies in the area [26] revealed the presence of *S. haematobium* infections in school children along this path as well as the presence of the intermediate host. In the Mount Cameroon area, human malaria is *meso*-endemic during the dry season but becomes hyper-endemic in the rainy season, with incidence peaking in July–October [27].

This study was conducted among primary school-aged children 4–14 years of both sexes whose parents consented to their participation in the study. Participation was voluntary and only children who had resided for at least three months in the study area took part in the study.

### Study design, sample size estimation and sampling

Following several control programmes and intervention strategies such as yearly school-based deworming of SAC with mebendazole/albendazole; chemotherapy with praziquantel in some identified regions in the country; nation-wide distribution of insecticide treated bed net (ITN) and LLINs; health education in schools and promoting hygiene and environmental sanitation (see Additional file 1: Figure S1) by the National programme for the control of schistosomiasis and intestinal helminthiasis and the National Malaria Control Programme (NMCP) through the Ministry Public Health, cross sectional studies were carried out in the urogenital schistosomiasis endemic focus in Muyuka Health District. This cross-sectional study which ran from March to June 2015 was a follow-up of a cross-sectional study carried out earlier [25].

The sample size for the study was determined using the formula *n* = *Z*^2^*pq*/*d*^2^ [28], where *n* was the sample size required; *Z* was 1.96, which is the standard normal deviate (for a 95% confidence interval, *CI*); *p* was 34.3%, the proportion of urogenital schistosomiasis prevalence reported previously in the area [25]; *q* was 1-*p*, the proportion of urogenital schistosomiasis negative; and *d* was 0.05, the acceptable error willing to be committed. The optimum minimum sample size obtained (346.3) was adjusted by 15% to a minimum of 398 in case of sample loss due to transportation difficulties and non-compliance by participants to provide all three samples. With respect to sampling, a representative sample from each primary school and study site was selected at random by balloting from each class accounting for the numbers above the calculated sample size. Likoko village with the highest number of primary schools had the highest number of study participants followed by Ikata and Bafia.

### Implementation of survey

Prior to the commencement of study, regular visits were made to the various study sites, to educate the inhabitants on the importance, benefits and protocol of the study. Informed consent forms were sent to parents/guardians through the pupils explaining the purpose and benefits of the study as well as the precautions taken to minimize risk. Children who presented signed consent forms were enrolled into the study and interviewed using a simple structured questionnaire to obtain information on both demography and factors that may be associated with the presence of the conditions. This was followed by clinical evaluation where weight, height and temperature were measured. The study involved the collection of venous blood, urine, and stool sample for haematological analysis, and microscopic detection of *S. haematobium* and STH eggs, respectively. Labelled blood and urine (placed on ice block) and stool samples preserved in 10% formalin were transported to the University of Buea Malaria Research Laboratory for further analysis.

### Data collection

#### Questionnaire administration

A semi-structured questionnaire was pre-tested on another community with similar characteristics as the study community and the questions were modified accordingly before being administered. This pre-tested questionnaire of approximately 5 min or less in length per respondent was administered to each participant by a trained research assistant with the aid of the teachers to obtain information on demography, personal hygiene and practices, health status and possible risk factors of *Plasmodium* and helminth infections as well as malnutrition and anaemia. The questionnaire was administered in English language and with a few exceptions in Pidgin English, the most widely spoken language in the area. The ages of participants were obtained from the school register.

#### Clinical evaluation

The axillary temperature was measured using a digital thermometer and a participant was considered febrile, if the body temperature was ≥ 37.5 °C. The height was measured to the nearest 0.1 cm using a graduated ruler of length 2 m. The body mass was measured to the nearest 0.5 kg using a mechanical scale of capacity 120 kg (KINLEE® model BR9310, Guangdong, China), and mid upper arm circumference (MUAC) was measured using a graduated tape. These measurements were used to calculate an array of anthropometric indices used as proxies for malnutrition: weight-for-age (WA: under-weight); height-for-age (HA: stunting) and weight-for height (WH: wasting). Anthropometric indices were computed as z-scores based on the WHO growth reference curves using the WHO AnthroPlus for personal computers manual [29]. Underweight was defined as a weight-for-age z (WAZ) score of <  − 2, wasting as a weight-for-height z (WHZ) score of <  − 2 and stunting as height-for-age z (HAZ) score of <  − 2. A child was identified as being malnourished if he or she scored <  − 2 in one of the anthropometric indices of WA, HA and WH indices [29].

#### Malaria parasite diagnosis and full blood count

Approximately 2 ml of venous blood was collected in ethylenediamine tetra-acetate tubes for malaria parasite detection and haematological analysis. Thick and thin blood films were prepared in situ, following standard operational protocol [30]. Thin blood films fixed in methanol and thick blood films were Giemsa stained and examined microscopically following standard procedures [30]. Slides were considered positive when asexual forms and/or gametocytes of any *Plasmodium* species were observed on the blood film. All the slides were read twice by two independent microscopists. Malaria parasite per µl of blood was determined by counting the number of parasites per 200 leukocytes and multiplying by the individuals white blood cell (WBC) count. Parasitaemia was classified as low (≤ 500 parasite/µl of blood), moderate (501–5000 parasites/µl of blood) and high (> 5000 parasites/µl of blood).

A complete blood count was ran using a Beckman Coulter counter (Urit 3300, Guilin Botest Medical Electronic Co., Ltd., Guilin, China) that automatically gave values for red blood cell (RBC), WBC and platelet counts, haemoglobin (Hb), haematocrit (Hct), mean cell volume (MCV), mean cell haemoglobin (MCH), mean cell haemoglobin concentration (MCHC) following the manufacturer’s instructions. The classification of anaemia (Hb concentration below the WHO reference values for age or gender) and its severity was done following WHO standards (mild anaemia = 100–109 g/L, moderate anaemia = 70–99 g/L and severe anaemia < 70 g/L) [25, 26]. Leucopenia was defined as WBC < 4.5 × 10^9^/L, hypochromasia as MCHC < 32 g/L [31], microcytosis as MCV < 73 fl and thrombocytopenia was defined as platelet count < 150 000/μl.

#### Urine analysis for haematuria and schistosome eggs

About 25 ml of midstream urine was collected into plastic screw cap vials after a brisk exercise between 10:00 am and 2:00 pm. Gross haematuria was determined by visual observation while micro haematuria was determined with the aid of reagent strips (Combistix) following the manufacturers guide (CYBOW™ 11 M a series of Health Mate Ref 0974, DFI Co., Ltd, Gomo-ro, 388–25 Korea). Following agitation, 10 ml of urine was drawn using a syringe and filtered through a polycarbonate membrane filter (STERLITECH corporation, Washington, USA). The filter membrane was examined microscopically for the presence of schistosome eggs as described by Cheesbrough [25]. Schistosome egg density was expressed as the number of eggs in 10 ml urine (eggs/10 ml) and the intensity of infection was categorised as either light (< 50 eggs/10 ml) or heavy infection (≥ 50 eggs/10 ml) [32, 33].

#### Faecal examination by Kato-Katz

Fresh stool samples were collected, smears were prepared and examined using the Kato-Katz thick smear method, as described by Cheesbrough [30]. Duplicate smears were prepared for each specimen using a 41.7 mg Kato-Katz template. Each slide was allowed to clear for 30 min, and then examined at 100 × total magnification within one hour of preparation to avoid missing hookworm eggs. The number of eggs counted per slide was multiplied by 24 to obtain the egg count per gram (epg) of faeces. As a quality control measure, all positive slides and 10% of randomly selected negative smears were re-examined by a third parasitologist who had no knowledge of the previous results. An average of the counts was utilised. Children were classified as having light (1–4999; 1–999 epg), moderate (5000–49 999; 1000–9999 epg) or heavy (≥ 50 000; ≥ 10 000 epg) infections for *A. lumbricoides* and *T. trichiura* respectively [34].

### Data analysis

Descriptive measures like the mean and standard deviation (SD), geometric means, frequencies, and proportions were used to summarize data. Polyparasitism was defined as infection with ≥ 2 of the parasites (*S. haematobium*/*Plasmodium*/STH). Differences in proportions between populations were compared using Chi (*χ*^2^) test. Geometric mean parasite density (GMPD) of *P. falciparum* and schistosome egg counts by age and sex were compared using analysis of variance (ANOVA) and the Student’s *t*-test, respectively and the correlation coefficient (*r*) was used to establish the relationship between the different parasite densities. Geometric means were computed for those positive only and the log transformed counts were used in the analysis. The adjusted odds ratio (a*OR*) in the multivariate analysis was used to see the strength of the association of the risk factors with infection. The 95% confidence interval (*CI*) was reported and *P*-values < 0.05 were considered indicative of statistical significance. The ability of a measurable morbidity to discriminate between infections and the diagnostic performance was evaluated using the receiver operating characteristics (ROC) curve analysis and the strength of agreement was determined by estimating the Kappa value. Kappa (к) was calculated using a Graphpad calculator [35] and the values interpreted as stated by Landis [36]. All data was analysed using IBM-Statistical Package for Social Science (SPSS) version 21 (IBM-SPSS Inc., Chicago, IL, USA).

### Ethical considerations

The study protocol was reviewed and approved by the Institutional Ethical Review Board hosted by the Faculty of Health Sciences, University of Buea (2014/243/UB/FHS/IRB) after administrative clearance from the South West Regional Delegation of Public Health and Basic Education were obtained. The population was sensitized in the various communities at the beginning of the study. Written informed consent was obtained from all parents/caregivers whose child/children participated in the study after explaining the purpose and benefits of their participation. Participation was totally voluntary, and a participant could opt out of the study at any time without any penalty. Participants who had malaria and or helminths were given first line treatment as recommended by the national treatment guideline policy for uncomplicated malaria (artesunate-amodiaquine) and helminths (praziquantel for urogenital schistosomiasis and mebendazole for STH).

## Results

### Characteristics of participants

Out of the 638 SAC with a mean (SD) age of 9.0 (2.1) years of both sexes (50.0% male and 50.0% female) examined, 386 (60.5%) were between 7 and 10 years old and majority (61.3%) were from the Likoko locality. The prevalence of stunting was 23.7% (95% *CI*: 20.5–27.1%) with significantly higher prevalence in males (29.8%, 95% *CI*: 25.0–35.0%) and children 11–14 years old (38.0%, 95% *CI*: 30.8–45.7%) than their respective equals. Overall, 4.7% (95% *CI*: 3.3–6.6%) of the children were overweight with significantly higher (*P* = 0.042) occurrences in males (6.3%, 95% *CI*: 4.1–9.5%) than females (3.1%, 95% *CI*: 1.7–5.7%). Similarly, the mean BAZ and the MUAC varied significantly with sex and age as shown in Table 1.

**Table 1:** Demographic and clinical characteristics of participants by sex and age

Parameter		Sex		*P*-value	Age group in years			Overall	*P*-value
		Male	Female		4–6	7–10	11–14		
		*Demographic*							
% (*n*)		50.0 (319)	50.0 (319)		14.7 (94)	60.5 (386)	24.8 (158)	100 (638)	
Mean age (SD)		8.9 (2.3)	9.0 (2.0)		5.6 (0.7)	8.7 (1.1)	11.7 (1.0)	9.0 (2.1)	
Site	Bafia	43.4 (43)	56.6 (56)	0.195	2.0 (2)	70.7 (70)	27.3 (27)	15.5 (99)	**< 0.001**
	Ikata	47.3 (70)	52.7 (78)		23.6 (35)	50.7 (75)	25.7 (38)	23.2 (148)	
	Likoko	52.7 (206)	47.3 (185)		14.6 (57)	61.6 (241)	23,8 (93)	61.3 (391)	
		*Nutritional indices*							
Mean height (SD) in cm		123.8 (12.0)	126.6 (12.2)	**0.004**	110.4 (10.3)	123.8 (8.5)	137.2 (9.1)	125.2 (12.1)	**< 0.001**
Mean weight (SD) in kg		27.7 (9.1)	28.1 (6.9)	0.510	21.3 (3.1)	26.5 (4.9)	35.2 (10.7)	27.9 (8.1)	** < 0.001**
Mean HAZ (SD)		− 1.2 (2.0)	− 1.0 (1.5)	**0.042**	− 0.23 (3.3)	− 1.13 (1.3)	− 1.63 (1.2)	− 1.1 (1.8)	** < 0.001**
Prevalence of Stunting (*n*)		29.8 (95)	17.6 (56)	** < 0.001**	10.6 (10)	21.0 (81)	38.0 (60)	23.7 (151)	** < 0.001**
Mean WAZ (SD)		− 0.05 (1.7)	− 0.12 (1.1)	0.560	0.80 (2.2)	− 0.30 (1.1)	− 0.68	− 0.1 (1.4)	** < 0.001**
Prevalence of underweight (*n*)		6.3 (20)	3.1 (10)	**0.042**	2.1 (2)	7.3 (28)	0.0 (0)	4.7 (30)	0.176
Mean WHZ (SD)		2.0 (2.1)	1.8 (2.6)	0.840	1.9 (2.2)	–	–	–	**–**
Prevalence of wasting (*n*)		0.3 (1)	0.3 (1)	0.582	2.1 (2)	–	–	–	**–**
Mean BAZ (SD)		0.67 (1.7)	0.36 (1.4)	**0.017**	1.3 (2.2)	0.46 (1.5)	0.16 (1.4)	0.52 (1.6)	** < 0.001**
Mean MUAC (SD)		18.4 (2.1)	19.0 (2.1)	**0.001**	17.0 (1.4)`	18.5 (1.7)	20.4 (2.3)	18.7 (2.1)	** < 0.001**
		*Schistosoma and malariometric indices*							
Anaemia prevalence (*n*)		74.9 (239)	73.7 (235)	0.393	85.1 (80)	73.8 (285)	69.0 (109)	74.3 (474)	**0.017**
Prevalence of fever (*n*)		23.3 (73)	19.7 (62)	0.160	20.9 (19)	23.8 (90)	16.5 (26)	21.5 (135)	0.166
*Schistosoma* MED (range)		17 (1–280)	22 (1–600)	0.397	22 (1–280)	15 (1–600)^a^	32 (1–450)^b^	20 (1–600)	0.071
Haematuria prevalence (*n*)		10.7 (34)	15.4 (49)	0.078	11.7 (11)	11.4 (44)	17.7 (28)	13.0 (83)	0.127
*Plasmodium* GMPD (range)		809 (140–33 250)	592 (71–12 721)	0.114	774 (158–18 090)	805 (110–33 250)^a^	410 (71–4763)^b^	687 (71–33 250)	**0.017**

*BAZ* body mass index (BMI)-for-age z-score, *GMPD* geometric mean parasite density, *HAZ* height for age z, *MED* mean egg density, *MUAC* mid upper arm circumference, *WAZ* weight for age z, *WHZ* weight for height z, *P* values in bold are statistically significant

^a,b,c^Means with disparate superscript are significantly different. Fever computed for 627 participants

Anaemia was prevalent in 74.3% (95% *CI*: 70.8–77.5%) of the children with significantly higher (*P* = 0.017) predominance in children 4–6 years old (85.1%) than those under; fever occurred in 21.5% (95% *CI*: 18.5–24.9%) and haematuria in 13.0% (95% *CI*: 10.6–15.8%) of the children. With respect to *S. haematobium*, the mean egg density (MED) was significantly higher in the 11–14 years old (32 eggs/10 ml of urine) than in those 7–10 years old (15 eggs/10 ml of urine). On the contrary, *Plasmodium* GMPD was significantly higher in those 7–10 years old (805 parasites/µl of blood) than in those 11–14 years (410 parasites/µl of blood) (Table 1). The mean haematological parameters varied significantly (*P* < 0.001) with age (Additional file 2: Table S1).

### Pattern of infection prevalence and intensity

Overall, the prevalence of *S. haematobium*, *Plasmodium* and STH was 25.1%, 24.9% and 5.0% respectively with significantly higher prevalence observed in children from Likoko (38.9%, 31.2%, 8.2%), those who did not use potable water (30.8%, 30.6%, 6.1%), practiced bathing in streams (29.9%, 28.4%, 6.3%) and those who openly defecated in the environment (27.1%, 26.4%, 5.2%) (Additional file 3: Table S2).

**Fig. 1:**
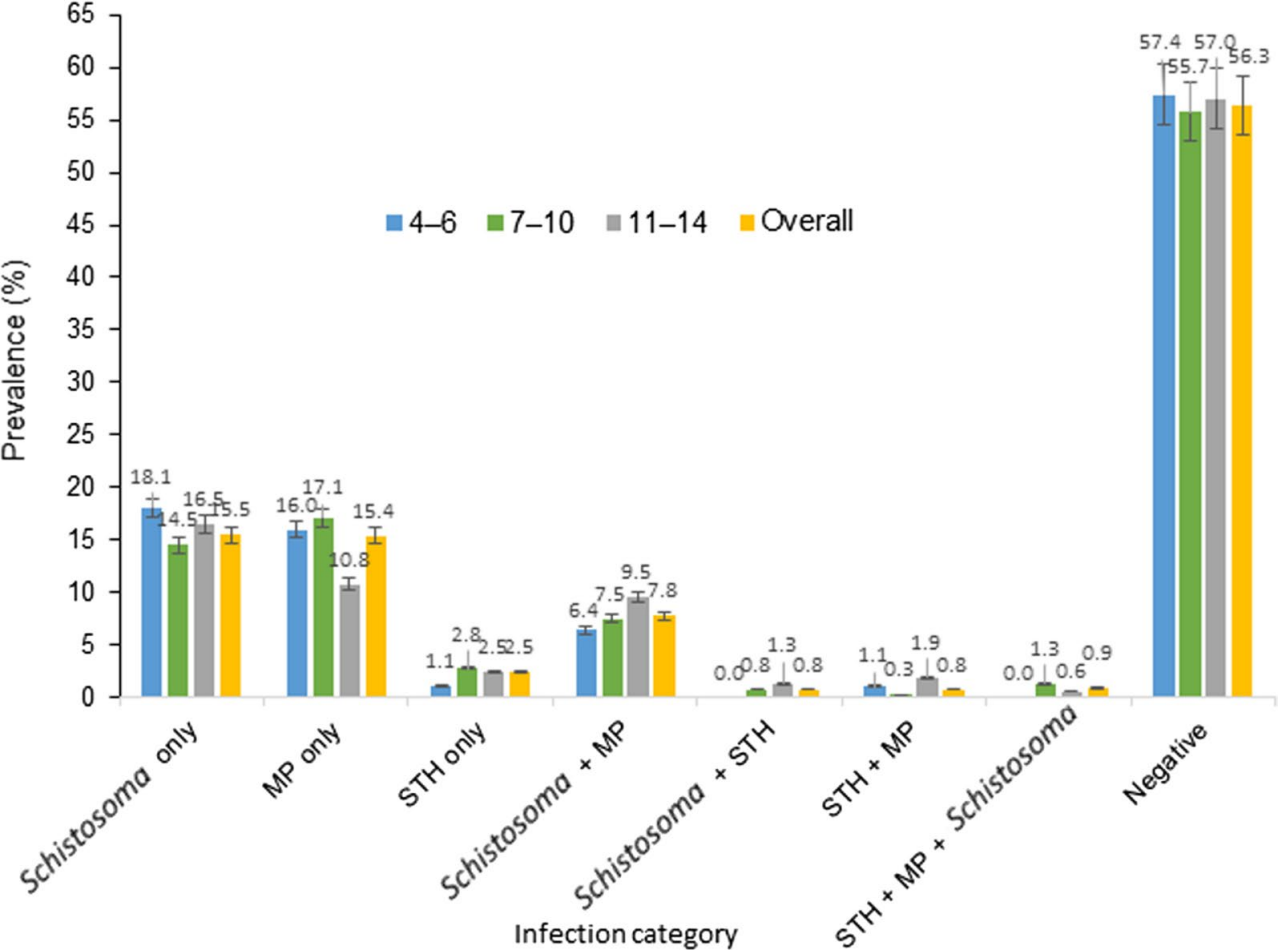
Prevalence of the different categories of infection by age. *MP* malaria parasite, *STH* soil-transmitted helminths. The different age groups in the study population are 4–6 years, 7–10 years and 11–14 years

The prevalence of single infection was 33.4% (95% *CI*: 29.8–37.1%) while polyparasitism occurred in 19.9% (95% *CI*: 17.0–23.2%) of the children. The pattern of infection prevalence by age is represented in Fig. 1. Single infection of *S. haematobium, P. falciparum* and STHs occurred in 15.5%, 15.4% and 2.5% of the children respectively with no significant differences with age. The prevalence of co-infections of *S. haematobium* + *P. falciparum* was 7.8%; *S. haematobium* + STH was 0.8%; *P. falciparum* + STH was 0.8% while, triple infection with *S. haematobium* + *P. falciparum* + STH was 0.9%.

Significantly higher preponderance of co-infection with *S. haematobium* + *Plasmodium* infection was observed in SAC who were females (10.0%, *P* = 0.039), from Likoko (12.3%, *P* < 0.001), did not use potable water (10.1%, *P* < 0.001), practiced bathing in stream (10.0%, *P* = 0.002) and carried out open defecation (8.8%, *P* = 0.018) when compared with their respective equivalents. Multiple parasite infections with *S. haematobium* + STH as well as *S. haematobium* + *P. falciparum* + STH were comparable across the different demographic and behavioural factors but for co-infection of *S. haematobium* + STH in males where a significantly higher (*P* = 0.025) prevalence was observed in males (1.6%) than females (0.0%) as shown in Table 2.

**Table 2:** Multiple parasite prevalence (95% *CI*) as influenced by demographic and behavioural factors

Characteristic	Category	*n*	*Schistosoma haematobium* + *Plasmodium*		*S. haematobium* + *P. falciparum* + STH		*S. haematobium* + STH	
			*n*	% (95% *CI*)	*n*	% (95% *CI*)	*n*	% (95% *CI*)
Sex	Male	319	18	5.6 (3.6–8.8)	3	0.9 (0.3–2.7)	5	1.6 (0.7–3.6)
	Female	319	32	10.0 (7.2–13.9)	3	0.9 (0.3–2.7)	0	0.0 (0.0–1.2)
	*P* value			**0.039**		1.00		**0.025**
Age group in years	4–6	94	6	6.4 (3.0–13.3)	0	0.0 (0.0–3.9)	0	0.0 (0.0–3.9)
	7–10	386	29	7.5 (5.5–10.6)	5	1.3 (0.6–3.0)	3	0.8 (0.3–2.3)
	11–14	158	15	9.5 (5.8–15.1)	1	0.6 (0.1–3.5)	2	1.3 (0.4–4.5)
	*P* value			0.628		0.455		0.545
Site	Bafia	99	0	0.0 (0.0–3.7)	0	0.0 (0.0–3.7)	0	0.0 (0.0–3.7)
	Ikata	148	2	1.4 (0.4–4.8)	0	0.0 (0.0–2.5)	0	0.0 (0.0–2.5)
	Likoko	391	48	12.3 (9.4–15.9)	6	1.5 (0.7–3.3)	5	1.3 (0.6–3.0)
	*P* value			** < 0.001**		0.48		0.204
Use of potable water (tap) source	Yes	142	0	0.0 (0.0–2.6)	0	0.0 (0.0–2.6)	0	0.0 (0.0–2.6)
	No	496	50	10.1 (7.7–13.0)	6	1.2 (0.0–2.6)	5	1.0 (0.4–2.3)
	*P* value			** < 0.001**		0.188		0.230
Bathing site	Home	140	2	1.4 (0.4–5.1)	0	0.0 (0.0–2.7)	0	0.0 (0.0–2.7)
	Stream	479	48	10.0 (7.6–13.0)	6	1.3 (0.6–2.7)	5	1.0 (0.5–2.4)
	Both	19	0	0.0 (0.0–16.8)	0	0.0 (0.0–16.8)	0	0.0 (0.0–16.8)
	*P* value			**0.002**		0.366		0.433
Distance to water source	Far (> 200 m)	308	23	7.5 (5.0–11.0)	5	1.6 (0.7–3.7)	1	0.3 (0.1–1.8)
	Near (< 200 m)	330	27	8.2 (5.7–11.6)	1	0.3 (0.1–1.7)	4	1.2 (0.5–3.2)
	Test			0.737		0.084		0.204
Nature of house	Plank	578	45	7.8 (5.9–10.3)	6	1.0 (0.5–2.3)	5	0.9 (0.4–2.0)
	Block	60	5	8.3 (3.6–18.1)	0	0 0 (0.0–6.0)	0	0.0 (0.0–6.0)
	Test			0.881		0.428		0.470
Open defecation behaviour	Yes	557	49	8.8 (6.7–11.4)	6	1.1 (0.5–2.3)	5	0.9 (0.4–2.1)
	No	81	1	1.2 (0.2–6.7)	0	0.0 (0.0–4.5)	0	0 0 (0.0–4.5)
	*P* value			**0.018**		0.348		0.392
BMI	Normal	535	42	7.9 (5.9–10.5)	5	0.9 (0.4–2.2)	4	0.7 (0.9–3.2)
	Thin	23	1	4.3 (0.7–21.0)	0	0.0 (0.0–14.3)	0	0.0 (0.0–14.3)
	Obese	75	5	6.7 (2.9–14.7)	1	1.3 (0.2–7.2)	1	1.3 (0.2–7.2)
	*P* value			0.783		0.844		0.692

*BMI* body mass index, *CI* confidence interval, *STH* soil-transmitted helminths, *P* values in bold are statistically significant

**Fig. 2:**
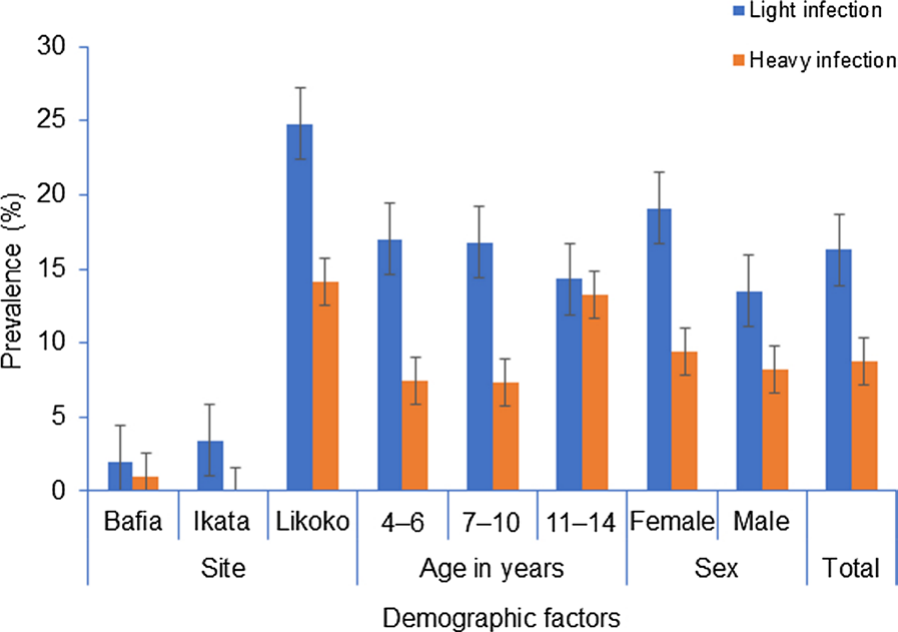
Infection intensity with *Schistosoma haematobium* as affected by site, age and sex

The prevalence of light and heavy infections with *S. haematobium* was 16.3% (104/638) and 8.8% (56/638) respectively. As shown in Fig. 2, no significant differences in prevalence of light and heavy infections with *S. haematobium* were observed with age and sex. With respect to site, the prevalence of both light and heavy infections was highest in SAC from Likoko (24.8% and 14.1% respectively) than those of Ikata and Bafia and the difference was statistically significant (*P* < 0.001).

**Fig. 3:**
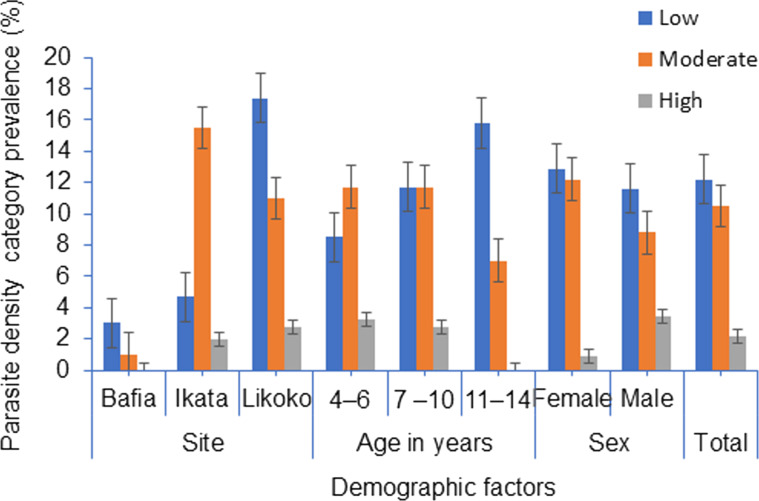
Malaria parasite density category prevalence by site, age and sex

Malaria parasite density category is represented in Fig. 3. Overall, low, moderate, and high parasite density was prevalent in 12.2% (78/638), 10.5% (67/638) and 2.2% (14/638) of the population, respectively. Statistically significant difference was observed with site (*χ*^2^ = 45.16, *P* < 0.001) with SAC from Likoko and Ikata having highest prevalence of low (17.4%) and moderate (15.5%) parasite density, respectively.

**Fig. 4:**
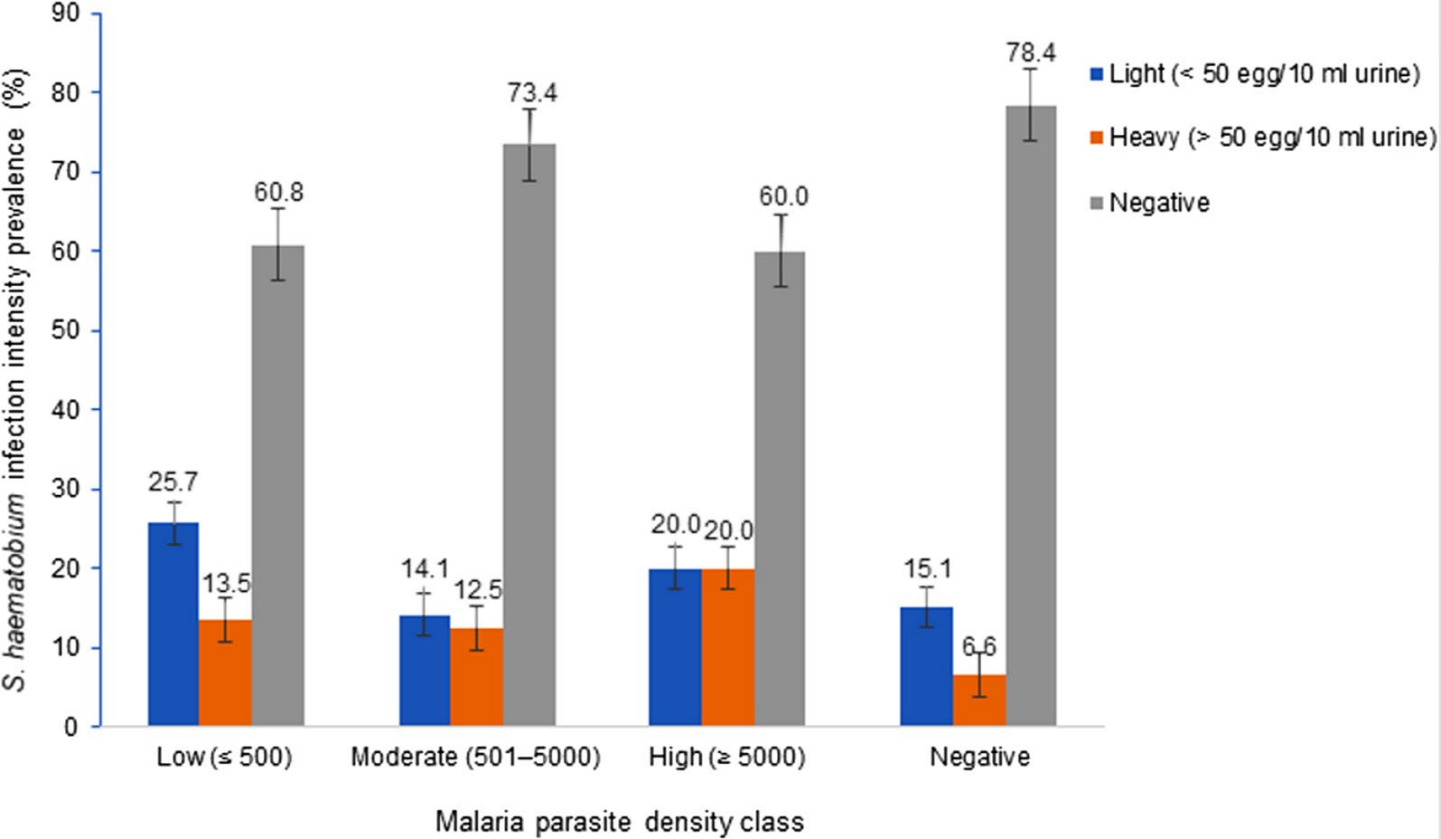
Prevalence of light and heavy intensity *Schistosoma haematobium* infections as affected by *Plasmodium falciparum* density

All infections with STHs were light [*A. lumbricoides* (4.1%, 26/638) and *T. trichuria* (1.9%, 12/638)] and occurred only in SAC of Likoko. After controlling for STH egg density, a negative trend in association (*r* = − 0.171, *P* = 0.784) was observed between *S. haematobium* egg density and malaria parasitaemia. However, significantly higher (χ^2^ = 14.83, *P* = 0.022) prevalence of heavy and light intensities of infection with *S. haematobium* was observed in children having *Plasmodium* infection of high (20.0%) and low densities (25.7%) respectively as shown in Fig. 4.

### Risk factors of polyparasitism

As shown in Table 3, the multivariate analysis revealed no significant demographic or behavioural factors associated with *S. haematobium* + *P. falciparum* + STH and *S. haematobium* + STH infections. Being female (a*OR* = 2.38, *P* = 0.009) was the only significant risk factor associated with *S. haematobium* + *Plasmodium* infection as they were 2.38 times at odds of having the co-infection. On the other hand, living in Ikata (a*OR* = 0.04, *P* < 0.001) and not practicing open defecation behaviour demonstrated significant protection against *S. haematobium* + *Plasmodium* co-infection.

**Table 3:** Risk factors of poly-infection with *Schistosoma haematobium*/*Plasmodium* and /STH

Variable	Category	*S. haematobium* + *Plasmodium*		*S. haematobium* + *P. falciparum*_+_ *STH*		*S. haematobium* + STH	
		a*OR* (95% *CI*)	*P*-value	a*OR* (95% *CI*)	*P* value	a*OR* (95% *CI*)	*P*-value
Sex	Female	2.38 (1.24–4.60)	**0.009**	1.35 (0.26–6.93)	0.716	4.8E-9 (0.0–*C*)	0.998
	Male	Reference		Reference		Reference	
Site	Bafia	1.01 × 10^–9^(0.0–*C*)	0.997	ND	ND	ND	ND
	Ikata	0.04 (0.01–0.18)	** < 0.001**	ND		ND	
	Likoko	Reference		Reference		Reference	
Open defecation behaviour	No	0.07 (0.01–0.49)	**0.008**	ND	ND	ND	ND
	Yes	Reference		Reference		Reference	

*aOR* adjusted odds ratio,* C* system missing*, CI* confidence interval, *STH* soil transmitted helminths, *ND* not determined as it is redundant in the model, *STH* soil-transmitted helminths, *P* values in bold are statistically significant

### Infection outcomes and clinical correlates

The most common clinical morbidity measured was anaemia (74.3%) followed by microcytosis (45.3%), malnutrition (26.5%), and the least was hypochromasia (1.6%). Apart from anaemia, the most common symptoms associated with *S. haematobium* infection were haematuria (46.5%), microcytosis (41.4%) and malnutrition (27.3%); *Plasmodium* sp. infection: microcytosis (45.9%), malnutrition (16.3%) and fever (14.4%), while for *Schistosoma* and *Plasmodium* co-infection was haematuria (54.0%), microcytosis (50.0%) and fever (36.0%) as shown in Table 4.

**Table 4:** Measured clinical morbidity by infection category

Infection category status	*n*	Prevalence of the different types of clinical morbidity							
		Fever % (*n*)	Anaemia % (*n*)	Malnutrition % (*n*)	Haematuria % (*n*)	Leucopenia % (*n*)	Thrombocytopenia % (*n*)	Microcytosis % (*n*)	Hypochromasia % (*n*)
All	638	21.2 (135)	74.3 (474)	26.5 (169)	13.0 (83)	1.9 (12)	10.8 (69)	45.3 (289)	1.6 (10)
*Schistosoma haematobium* only	99	20.2 (20)	80.8 (80)	27.3 (27)	46.5 (46)	4.0 (4)	9.1 (9)	41.4 (41)	3.0 (3)
MP Only	98	14.3 (14)	79.6 (78)	16.3 (16)	1.0 (1)	2.0 (2)	9.2 (9)	45.9 (45)	1.0 (1)
STH Only	16	25.0 (4)	68.8 (11)	37.5 (6)	0.0 (0)	0.0 (0)	6.3 (1)	43.8 (7)	0.0 (0)
*Schistosoma* + MP	50	36.0 (18)	82.0 (41)	22.0 (11)	54.0 (27)	6.0 (3)	24.0 (12)	50.0 (25)	2.0 (1)
*Schistosoma* + STH	5	20.0 (1)	40.0 (2)	20.0 (1)	80.0 (4)	0.0 (0)	0.0 (0)	0.0 (0)	0.0 (0)
*Schistosoma* + MP + STH	6	0.0 (0)	100 (6)	50.0 (3)	83.3 (5)	0.0 (0)	0 0 (0)	50.0 (3)	0.0 (0)
*Schistosoma*/MP/STH	66	28.7 (19)	80.3 (53)	25.8 (17)	54.5 (36)	4.5 (3)	19.7 (13)	48.5 (32)	1.5 (1)
Negative	359	21.7 (78)	70.2 (252)	28.7 (103)	0.0 (0)	0.8 (3)	10.3 (37)	45.7 (164)	1.4 (5)

*MP* malaria parasite, *STH* soil-transmitted helminths

Analysis of the suitability of the clinical signs measured to determine the symptoms of the disease revealed the specificity of haematuria in predicting *S. haematobium* infection was 100% (95% *CI*: 98.9–100%), with a sensitivity of 46.5% (95% *CI*: 37.0–56.2%) and a moderate agreement (*к* = 0.576) with microscopy, while the sensitivity and specificity of presumptive use of anaemia and microcytosis were 80.8% and 29.8% vs 41.4% and 54.3% with a slight and no agreement respectively with microscopy. In relation to *Plasmodium* infection the sensitivity and specificity of presumptive use of anaemia and microcytosis to predict infection was 79.6% and 29.8 vs 45.9% and 54.3% with a slight agreement (*к* = 0.051 and *к* = 0.002) respectively with microscopy as shown in Table 5.

**Table 5:** Diagnostic characteristic of measured clinical morbidity by infection category

Diagnostic characteristic		*Schistosoma haematobium*			*Plasmodium* sp.	
		Haematuria	Anaemia	Microcytosis	Anaemia	Microcytosis
Sensitivity	% (95% *CI*)	46.5 (37.0–56.2)	80.8 (72.0–87.4)	41.4 (32.2–51.3)	79.6 (70.6–86.4)	45.9 (36.3–55.8)
Specificity	% (95% *CI*)	100 (98.9–100)	29.8 (25.3–34.7)	54.3 (49.2–59.4)	29.8 (25.3–34.7)	54.3 (49.2–59.4)
Positive likely ratio	(95% *CI*)	ND	1.15 (1.02–1.29)	0.91 (0.70–1.18)	1.13 (1.01–1.28)	1.01 (0.79–1.28)
Negative likely ratio	(95% *CI*)	0.54 (0.45–0.65)	0.64 (0.42–0.99)	1.08 (0.89–1.31)	0.69 (0.45–1.04)	1.00 (0.81–1.22)
Diagnostic odds ratio	(95% *CI*)	ND	1.79 (1.03–3.10)	0.84 (0.54–1.32)	1.66 (0.96–2.84)	1.01 (0.65–1.58)
ROC	*AUC*	0.698	0.539	0.477	0.531	0.504
	SE	0.033	0.031	0.031	0.031	0.032
	95% *CI*	0.633–0.763	0.479–0.598	0.415–0.539	0.471–0.592	0.441–0.566
Kappa (*к*)	Value	0.576	0.057	− 0.031	0.051	0.002
	SE	0.050	0.026	0.040	0.026	0.040
	95% *CI*	0.479–0.674	0.007–0.107	− 0.110–0.048	− 0.00–0.100	− 0.077–0.081
	Agreement	Moderate	Slight	No agreement	Slight	Slight

*AUC* area under the ROC curve, *CI* confidence interval, *ND* not determine as it is redundant, *SE* standard error, *ROC* receiver operating characteristics

Overall, the most common unmeasurable clinical outcome reported was fever in the past 3 days (51.7%), followed by lower abdominal pain (43.4%) and fever in the last 3 months (40.0%). As shown in Table 6, SAC who had single infection with *S. haematobium* were 1.83 times and 1.68 times at odds of reporting fever in the last 3 days (a*OR* = 1.83, *P* = 0.015) and headaches (a*OR* = 1.68, *P* = 0.045) respectively. Similarly, co-infection with *S. haematobium* and malaria parasite was significantly associated with threefold odds of history of fever in the last three days (a*OR* = 3.02, *P* = 0.001) and in addition 2.84 times at odds of having lower abdominal pain (a*OR* = 2.84, *P* = 0.002). While SAC with *S. haematobium* + STH and those with malaria parasite and STH infections were 3.32 times and 2.12 times at odds of reporting diarrhoea and vomiting respectively, the risk was not statistically significant.

**Table 6:** Association between self-reported outcomes and infection category

Outcome	% (*n*)	Infection category	Adjusted *OR* (95% *CI*)	*P*-value
Fever in last 3 months	31.3 (5)	STH only	0.49 (0.15–1.54)	0.221
	56.1 (55)	MP only	1.32 (0.82–2.13)	0.258
	57.6 (57)	*Schistosoma* only	1.16 (0.71–1.89)	0.564
	80.0 (4)	STH + MP	5.13 (0.53–50.10)	0.160
	62.0 (31)	*Schistosoma* + MP	1.02 (0.52–2.01)	0.954
	80.0 (4)	*Schistosoma* + STH	2.91 (0.28–30.45)	0.372
	100 (6)	*Schistosoma* + MP + STH	ND	ND
Fever in last 3 days	31.3 (5)	STH Only	1.13 (0.36–3.56)	0.834
	43.9(43)	MP only	1.49 (0.92–2.41)	0.102
	49.5 (49)	*Schistosoma* only	1.83 (1.13–2.96)	**0.015**
	40.0 (2)	STH + MP	0.88 (0.17–6.12)	0.900
	64.0 (32)	*Schistosoma* + MP	3.02 (1.55–5.89)	**0.001**
	60.0 (3)	*Schistosoma* + STH	2.26 (0.34–14.90)	0.398
	66.7 (4)	*Schistosoma* + MP + STH	2.59 (0.44–15.34)	0.293
Lower abdominal pain	31.3 (5)	STH Only	0.87 (0.28–2.68)	0.811
	41.8 (41)	MP only	1.02 (0.64–1.64)	0.929
	49.5 (49)	*Schistosoma* only	1.37 (0.86–2.20)	0.186
	40.0 (2)	STH + MP	0.84 (0.13–5.66)	0.858
	68.0 (34)	*Schistosoma* + MP	2.84 (1.46–5.50)	**0.002**
	60.0 (3)	*Schistosoma* + STH	1.62 (0.25–10.34)	0.612
	33.3 (2)	*Schistosoma* + MP + STH	0.41 (0.07–2.55)	0.342
Headache	31.3 (5)	STH only	2.00 (0.69–6.16)	0.228
	23.5 (23)	MP only	0.95 (0.55–1.163)	0.853
	35.4 (35)	*Schistosoma* only	1.68 (1.02–2.78)	**0.041**
	20.0 (1)	STH + MP	0.78 (0.08–7.30)	0.827
	38.0 (19)	*Schistosoma* + MP	1.69 (0.87–3.26)	0.118
	40.0 (2)	*Schistosoma* + STH	1.56 (0.24–10.18)	0.640
	33.3 (2)	*Schistosoma* + MP + STH	1.27 (0.22–7.42)	0.788
Diarrhoea	0 0 (0)	STH only	ND	ND
	17.3 (17)	MP only	0.84 (0.46–1.57)	0.591
	12.1 (12)	*Schistosoma* only	0.59 (0.29–1.17)	0.129
	0.0 (0)	STH + MP	ND	ND
	10.0 (5)	*Schistosoma* + MP	0.46 (0.17–1.24)	0.123
	40.0 (2)	*Schistosoma* + STH	3.32 (0.53–20.83)	0.201
	0.0 (0)	*Schistosoma* + MP + STH	ND	ND
Vomiting	6.3 (1)	STH only	0.62 (0.07–4.96)	0.653
	15.3 (15)	MP only	0.94 (0.49–1.80)	0.845
	9.1 (9)	*Schistosoma* only	0.54 (0.25–1.18)	0.121
	20.0 (1)	STH + MP	2.12 (0.21–21.43)	0.524
	10.0 (5)	*Schistosoma* + MP	0.55 (0.20–1.52)	0.248
	0.0 (0)	*Schistosoma* + STH	ND	ND
	16.7 (1)	*Schistosoma* + MP + STH	1.67 (0.17–16.70)	0.662

*MP* malaria parasite, *ND* not determined as it was redundant in the model, *STH* soil-transmitted helminths. *P* values in bold are statistically significant

## Discussion

Concomitant *Plasmodium* and helminth (*S. haematobium* and STHs) infections are common in parts of South-West Cameroon [8, 37, 38], due to geographic overlap of climatic and socio-economic conditions that support survival of the malaria parasite vectors, egg and larval development of STHs and *Schistosoma* sp. intermediate snail hosts. Monitoring the effectiveness of intervention programs in reducing disease prevalence and examining the roles behaviour, demography, and nutritional status play in the co-occurrence of parasitic infection as well as attributing morbidity-related outcomes is heartened. Following the implementation of control strategies and interventions such as MDA of albendazole to SAC, praziquantel for urogenital schistosomiasis in some endemic areas and the free distribution of LLIN to communities in the Mount Cameroon area [20, 22, 23], we determined the prevalence and determinants of polyparasitism and evaluated the outcomes and clinical correlates of infections in SAC living in the urogenital schistosomiasis endemic foci of Bafia, Ikata and Likoko.

Polyparasitism occurred in 19.9% of the children although the prevalence of single infection was more common with similar occurrence of *S. haematobium* and *P. falciparum* infection. This polyparasitism prevalence in SAC is higher than 7.6% observed in Mbam and Inoubou Division, within the Centre Region of Cameroon [39], 11.2% in Ghana [40] and lower than the 30% and 28% observed in Kenya [41, 42]. When compared with previous studies in the same locality [25, 43], a decline in infections with *S. haematobium* and *P. falciparum* following MDA was observed in SAC. However, the prevalence of polyparasitism is likely to remain a significant public health problem in the Ikata-Likoko area where environmental (streams near homes, high rainfall) and socio-economic (farming and fishing activities, inadequate health care services, low level of education) characteristics are likely to favour the transmission of these infections. Again, while the national control strategy for helminth infection in SAC may curb transmission, infected individuals not included in the programme are likely to serve as a source of re-infection due to their common exposure to snail infested streams serving the communities.

The predominance of *S. haematobium* and *P. falciparum* (7.8%) co-infection when compared with *S. haematobium* and STH (0.8%), MP and STH (0.8%) and *S. haematobium, P. falciparum* and STH (0.9%) is not unusual. This may be attributed to the significant decline in STH infections in the Mount Cameroon area following the school-based deworming (SBDW) strategy with mebendazole adapted by Cameroon in 2004 and has been implemented annually since 2007 in both enrolled and unenrolled children [44, 45]. This *S. haematobium* and *P. falciparum* co-infection is of public health importance as the prevalence is higher than the 0.9% observed in Accra Ghana [46], comparable to the 9.0% in Gabon [47], lower than 10.9% and 13.6% reported in Mvomero-Tanzania and West Region of Cameroon respectively [48, 49] and within the 2.84% to 57.1% range reported in Africa [16, 50, 51].

Findings from the univariate analysis revealed being female, site (Likoko), children who did not use potable water, usually bathed in streams and carried out open defecation were more likely to have *S. haematobium* and *Plasmodium* co-infection with interchangeable factors affecting the prevalence of *P. falciparum* and STH. Similar factors have been reported elsewhere [9, 47, 52]. However, the multivariate analysis demonstrated being female was the only significant risk factor with 2.38 times likelihood of having the *S. haematobium* and *Plasmodium* co-infection. This is not surprising as females spend more contact time in infested streams washing clothes, playing, swimming, and when bathing hence, the likelihood to be re-infected after treatment is higher [53, 54]. Albeit *S. haematobium* and *P. falciparum* have distinct transmission patterns, findings from the study (Additional file 3: Table S3) demonstrated similar drivers of the infections. This probably asserts the influence of environmental and host factors on the epidemiological and geographical patterns of infections and diseases [55]. Hence, in addition to the existing control measures, sustainable multidisciplinary intervention that integrates preventive chemotherapy with education on water, sanitation and hygiene (WASH), provision of potable water supply to communities, appropriate faecal disposal methods and improvement in health facilities and care is desired to reduce the burden of parasitic infections.

Worthy of note is the abundance of light infections with *S. haematobium* and low-density malaria parasite infections observed. In addition, all infections with STHs (*A. lumbricoides* and *T.s trichuria*) were light and occurred mostly in SAC of Likoko area. The consequences of the absence of potable water supply and an integrated health centre in the Likoko community is undoubtedly demonstrated here by the presence and high occurrence of all the identified parasites, suggestive of contaminated environment than the other localities. The high prevalence of light infection is consistent with similar studies in Nigeria, Malawi and Ghana [46, 53, 56]. Light infections can occur in populations previously targeted for schistosomiasis control [57] on the other hand, high prevalence of heavy intensity infection suggestive of long-term transmission and attributable to poor sanitation and water supply facilities have also been reported [58]. Most likely, the MDA with an anti-helminthic each year and the ineffective use of LLINs were not successful in preventing reinfections but probably aided in maintaining lower grade parasite intensities in the population.

Low levels of parasite loads represent chronic parasite infections which may play a major role in clinical morbidity [59]. The effects of polyparasitism which are often clinically inapparent may lead to multiple morbidities. While no significant antagonistic interaction between *S. haematobium* and *P. falciparum* densities was observed nevertheless, co-infections may exacerbate disease symptoms due to one of the pathogens. Observations from the study revealed anaemia as the most common (74.3%) clinical morbidity measured and its occurrence was exacerbated in co- and triple-infection with *Plasmodium* and helminths in line with Nyarko et al. [46]. Furthermore, a slight agreement in sensitivity and specificity of anaemia with microscopy in predicting the presence of both *S. haematobium* and *P. falciparum* infections was proven. While the spectrum of anaemia is broad and complex in resource-limited settings, these findings assert the significant contributions of urogenital schistosomiasis and malaria to the burden of anaemia in endemic areas accentuated by several studies [51, 60, 61] and could be a valuable diagnostic marker of both infections given its sensitivity.

Morbidity associated with urogenital schistosomiasis is caused by granulomatous reactions formed in response to egg deposition in the walls of the urinary tract, triggering inflammatory reaction, haematuria, proteinuria, fibrosis with ensuing obstruction and bladder carcinogenesis [62, 63]. Haematuria or bloody urine is a classic sign of urogenital schistosomiasis and findings from the study revealed an overall prevalence of 13.0%. This is lower than 16.6% observed in SAC in Northern Angola [64]. Haematuria was the second most common morbidity associated with urogenital schistosomiasis with 100% specificity, sensitivity of 46.5% and a moderate kappa agreement with microscopy in predicting the presence of the infection. The high specificity and low sensitivity observed is not atypical even though the sensitivity is lower than the 65% reported in populations with lower intensity infections [65]. Nonetheless, this is congruent with synthesis of previous findings that highlight dipstick sensitivity to decrease while specificity increases when compared to dipstick performance in high prevalence areas. This lends support for the need of a combination of diagnostic tools including clinical criteria as light and old infections may be missed by microscopy [66].

Other morbidities of significance observed in the study were microcytosis (45.3%) and malnutrition (26.5%). The prevalence of malnutrition (24.4%), with the most common being stunting, is comparable to those of SAC in localities close by [67] and lower than the 29.7% in SAC in rural Senegal [68]. Observation from the study showed a general inclination of SAC with *P. falciparum* to have predominance of microcytosis while those with *S. haematobium* had a higher occurrence of malnutrition. Unlike the increase in prevalence of microcytosis observed in *P. falciparum* and *S. haematobium* co-infection, increase in malnutrition prevalence was observed in triple infections of *S. haematobium, P. falciparum* and STH only. Although the directionality of causality of these morbidities are not very specific, microcytosis have been previously associated with protection against erythrocytic stage *Plasmodium* infection and severe malarial anaemia [69, 70]. On the other hand, the growth faltering and malnutrition attributed to urogenital schistosomiasis has been linked to chronic anti parasite inflammation which persists during childhood [68, 71–73].

A history of fever in the past 3 days was the most common unmeasurable clinical outcome reported while fever pervasiveness was lower. In addition, co-infection with *S. haematobium* and malaria parasite was significantly associated with threefold odds of history of fever in the last three days. Fever is a non-specific marker of infection that is often considered as a symptom of malaria in endemic areas. It results from endogenous pyrogen molecules activities, notably pro-inflammatory cytokine tumour necrosis factor (TNF)-α. However, *S. haematobium* infection could further augment anti-inflammatory responses induced by asymptomatic *P. falciparum* infection reducing the risk of fever probably accounting for the low occurrence of fever in the population [47, 74]. Other common morbidities of significance reported associated with co-infections include lower abdominal pain, diarrhoea and vomiting.

While the sensitivity and specificity of the diagnostic test varies according to the number of stool samples provided, the Kato-Katz technique has been reported to perform with reasonable accuracy with one day’s stool collection for *A. lumbricoides* and *T. trichiura* [75]. Notwithstanding, the use of a single stool and urine sample for the detection of helminth infection is a limitation in the study as this may have led to underestimation of the prevalence of polyparasitism as well as the intensities of the infections considering the variation in day to day excretion of eggs of some of these parasites. Other intestinal parasites may have gone undetected due to the limited sensitivity of the Kato-Katz technique in detecting other parasites. Despite this underestimation, we consider the data meaningful to reveal implications on disease-related outcome and clinical correlates.

Furthermore, despite the implementation of the various control measures and strategies against infection with *S. haematobium*, *Plasmodium* and STH as outlined earlier, the continued public health threat posed by these infections demonstrate the inadequacies of the measures. There is a need for an upscale in community-based context-specific complementary interventions strategies such as proper environmental management and provision of potable water accessible and affordable to all especially in newly identified foci for urogenital schistosomiasis to supplement the existing national policies.

## Conclusions

Polyparasitism is a public health problem in the Ikata-Likoko area in Muyuka even though single infection with either *Plasmodium* or *S. haematobium* was more common. Similar behavioural and environmental drivers of co-infections were observed with females most at risk hence, more sustainable, multidisciplinary, aggressive intervention control strategy is needed. Anaemia was the most common clinical morbidity measured and its occurrence was exacerbated in co- and triple-infection hence, anaemia could be a valuable diagnostic marker of both urogenital schistosomiasis and malaria given its sensitivity in resource-limited endemic areas. Haematuria was specific to urogenital schistosomiasis while there was a general inclination of SAC with *P. falciparum* to have predominance of microcytosis while those with *S. haematobium* had a higher occurrence of malnutrition. While lower abdominal pain, diarrhoea and vomiting were commonly reported, fever and principally a history of it is of value in predicting polyparasitism.

## Supplementary Information

**Additional file 1: Figure S1.** Relevant urogenital schistosomiasis, malaria and soil-transmitted helminth control in Cameroon and study area. First school-based deworming with mebendazole/albendazole in primary school children commenced in 2007 and proceeded yearly. This was extended to secondary school children in 2012. Systemic distribution of ITN to household commenced in 2006 through 2011, free treatment of children with uncomplicated malaria was instituted in 2010 and free diagnosis of malaria parasite in publics sector commenced in 2012.

**Additional file 2: Table S1.** Mean (SD) haematological parameters of participants by sex and age. Children 4–6 years had significantly lower mean Hb [102 (15) g/L], Hct [29.2 (4.3) %), RBC [4.1 (6.0) × 10^12^/L], MCV [71.6 (5.4) fl] and MCH [24.7 (1.9) pg], while the mean RDW-CV was highest [13.0 (1.5) %] when compared respectively. In relation to sex the only significant difference (*P* = 0.028) was observed in mean MCV with males having a lower value [72.9 (6.1) fl] than females [73.9 (6.1) fl].

**Additional file 3: Table S2.** Prevalence (95% *CI*) of infection with *Schistosoma haematobium*, *Plasmodium* and STH by demographic and behavioural factors. When compared with their peers, the prevalence of *S. haematobium* infection was significantly higher in females than males while that of *P. falciparum* and STH was comparable.

## Data Availability

All datasets generated and analysed during the current study are presented in the paper and supporting information files.

## Abbreviations

a*OR*: Adjusted odds ratio
*CI*: Confidence interval
GMPD: Geometric mean parasite density
Hct: Haematocrit
Hb: Haemoglobin
IHC: Integrated Health Centre
ITN: Insecticide treated bed net
MCH: Mean cell haemoglobin
MCHC: Mean cell haemoglobin concentration
MCV: Mean cell volume
MED: Mean egg density
MUAC: Mid upper arm circumference
LLINs: Long-lasting insecticidal nets
MDA: Mass drug administration
NTD: Neglected tropical diseases
PC: Preventive chemotherapy
RBC: Red blood cell
ROC: Receiver operating characteristics
SAC: School-aged children
SD: Standard deviation
STH: Soil-transmitted helminths
WBC: White blood cell
WHO: World Health Organisation
