# Supplementary material for: Polyparasitism with Schistosoma haematobium, Plasmodium and soil-transmitted helminths in school-aged children in Muyuka–Cameroon following implementation of control measures: a cross sectional study
Source: Infect Dis Poverty. 2021 Feb 17;10:14. doi: 10.1186/s40249-021-00802-x (PMC7890808; doi:10.1186/s40249-021-00802-x)
Supplement: Supplementary file 1 — Additional file 1: Figure S1. Relevant urogenital schistosomiasis, malaria and soil-transmitted helminth control in Cameroon and study area. First school-based deworming with mebendazole/albendazole in primary school children commenced in 2007 and proceeded yearly. This was extended to secondary school children in 2012. Systemic distribution of ITN to household commenced in 2006 through 2011, free treatment of children with uncomplicated malaria was instituted in 2010 and free diagnosis of malaria parasite in publics sector commenced in 2012. [file 40249_2021_802_MOESM1_ESM.pdf]

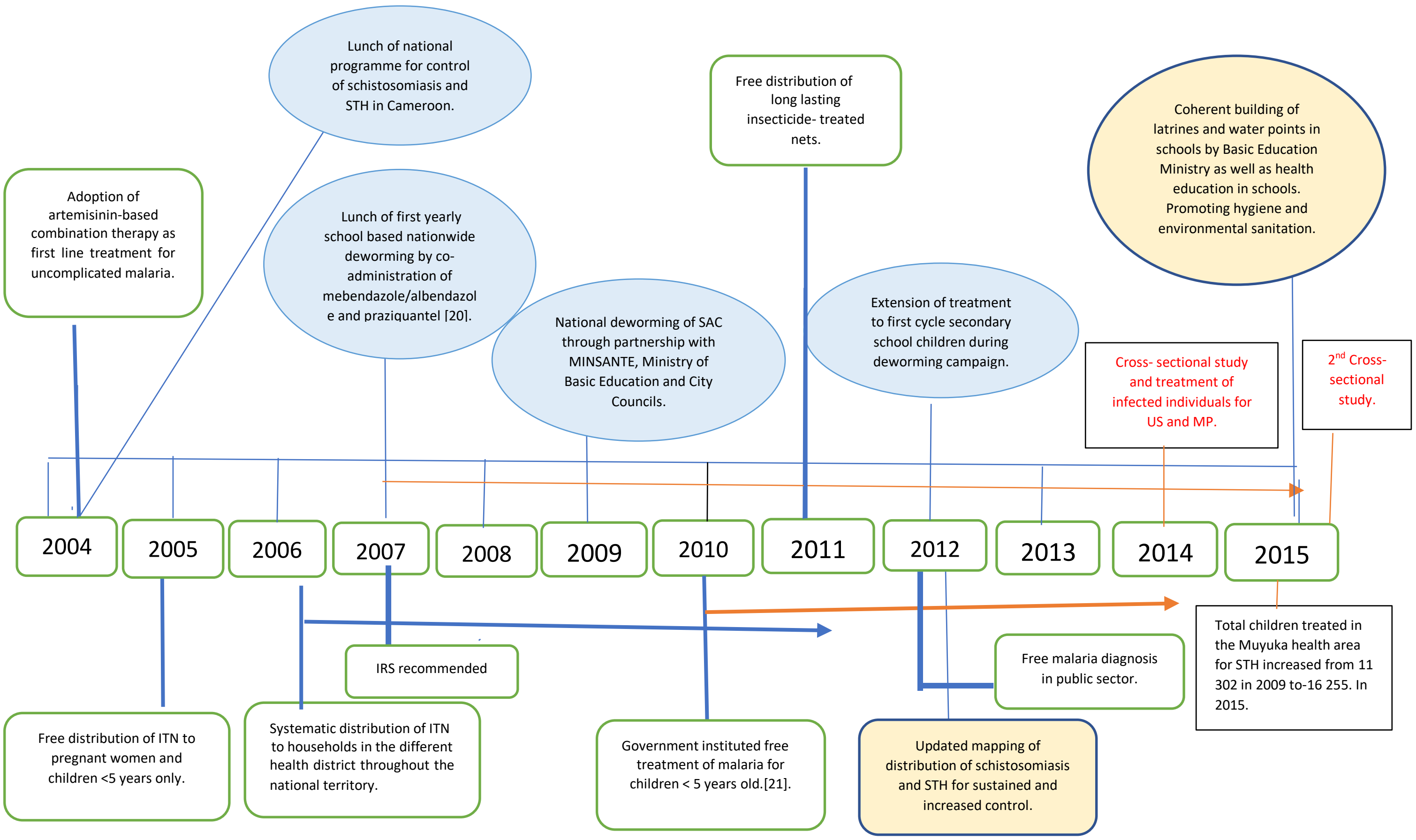

Additional file 1. Relevant urogenital schistosomiasis, malaria and soil-transmitted helminth control in Cameroon and study area
