# Supplementary material for: Polyparasitism with Schistosoma haematobium, Plasmodium and soil-transmitted helminths in school-aged children in Muyuka–Cameroon following implementation of control measures: a cross sectional study
Source: Infect Dis Poverty. 2021 Feb 17;10:14. doi: 10.1186/s40249-021-00802-x (PMC7890808; doi:10.1186/s40249-021-00802-x)
Supplement: Supplementary file 2 — Additional file 2: Table S1. Mean (SD) haematological parameters of participants by sex and age. Children 4–6 years had significantly lower mean Hb [102 (15) g/L], Hct [29.2 (4.3) %), RBC [4.1 (6.0) × 1012/L], MCV [71.6 (5.4) fl] and MCH [24.7 (1.9) pg], while the mean RDW-CV was highest [13.0 (1.5) %] when compared respectively. In relation to sex the only significant difference (P = 0.028) was observed in mean MCV with males having a lower value [72.9 (6.1) fl] than females [73.9 (6.1) fl]. [file 40249_2021_802_MOESM2_ESM.docx]

**Additional file 2. Mean haematological parameters of participants by sex and age**

| Parameter | Sex | | Test | Age group in years | | | Overall | Test |
| --- | --- | --- | --- | --- | --- | --- | --- | --- |
|  | Male | Female |  | 4–6 | 7–10 | 11–14 |  |  |
| Hb in g/L | 106 (14) | 107 (13) | 0.260 | 102 (15)^a^ | 106 (13)^b^ | 112 (12)^c^ | 107 (14) | **< 0.001** |
| Hct in % | 30.3 (4.0) | 30.9 (3.6) | 0.053 | 29.2 (4.3)^a^ | 30.4 (3.7)^b^ | 32.0 (3.4)^c^ | 30.6 (3.8) | **< 0.001** |
| WBC x 10 ^9^/L | 9.7 (4.1) | 9.6 (5.6) | 0.794 | 10.2 (4.5) | 9.7 (5.4) | 9.1 (3.2) | 9.6 (4.9) | 0.217 |
| RBC x 10^12^/L | 4.2 (5.2) | 4.2 (4.7) | 0.677 | 4.1 (6.0)^a^ | 4.2 (4.8)^ab^ | 4.3 (4.4)^c^ | 4.2 (5.0) | **0.002** |
| Lymphocyte x 10^9^L | 4.2 (2.8) | 4.0 (2.5) | 0.378 | 4.6 (2.7) | 4.1 (2.9) | 3.9 (2.1) | 4.1 (2.7) | 0.157 |
| MCV in fL | 72.9 (6.1) | 73.9 (6.1) | **0.028** | 71.6 (5.4)^a^ | 73.3 (6.2)^b^ | 74.6 (6.2)^c^ | 73.4 (6.1) | **0.001** |
| MCH in pg | 25.4 (2.4) | 25.6 (2.1) | 0.300 | 24.7 (1.9)^a^ | 25.5 (2.4)^b^ | 25.9 (2.1)^bc^ | 25.5 (2.2) | **< 0.001** |
| MCHC in g/L | 35.0 (2.4) | 34.7 (1,7) | 0.068 | 34.8 (2.6) | 34.9 (2.0) | 34.8 (1.8) | 34.9 (2.1) | 0.711 |
| Platelet x 10^9^L | 276.7 (113.8) | 280.7 (110.2) | 0.653 | 292.7 (156.2) | 278.2 (110.4) | 271.4 (84.9) | 278.7 (111.9) | 0.340 |
| RDW-CV% | 12.7 (1.4) | 12.3 (1.3) | < 0.001 | 13.0 (1.5)^a^ | 12.5 (1.3)^b^ | 12.3 (1.2)^bc^ | 12.5 (1.3) | **< 0.001** |

P-values in bold are statistically significant. ^a, b, c^ Means with disparate superscript are significantly different. Fever computed for 627 participants.
