# Supplementary material for: Polyparasitism with Schistosoma haematobium, Plasmodium and soil-transmitted helminths in school-aged children in Muyuka–Cameroon following implementation of control measures: a cross sectional study
Source: Infect Dis Poverty. 2021 Feb 17;10:14. doi: 10.1186/s40249-021-00802-x (PMC7890808; doi:10.1186/s40249-021-00802-x)
Supplement: Supplementary file 3 — Additional file 3: Table S2. Prevalence (95% CI) of infection with Schistosoma haematobium, Plasmodium and STH by demographic and behavioural factors. When compared with their peers, the prevalence of S. haematobium infection was significantly higher in females than males while that of P. falciparum and STH was comparable. [file 40249_2021_802_MOESM3_ESM.docx]

**Additional file 3. Prevalence (95% *CI*) of infection with *Schistosoma haematobium*, *Plasmodium* and STH by demographic and behavioural factors**

| Characteristic | Category | *n* | *S. haematobium* | | *Plasmodium* | | STH | |
| --- | --- | --- | --- | --- | --- | --- | --- | --- |
|  |  |  | n | % (95% *CI*) | N | % (95% *CI*) | n | % (95% *CI*) |
| All | All | 638 | 160 | 25.1 (21.9–28.6) | 159 | 24.9 (21.7–28.4) | 32 | 5.0 (3.6–7.0) |
| Sex | Male | 319 | 69 | 21.6 (17.5–26.5) | 76 | 23.8 (19.5–28.8) | 21 | 6.6 (4.4–9.9) |
|  | Female | 319 | 91 | 28.5 (23.9–33.7) | 83 | 26.0 (21.5–31.1) | 11 | 3.5 (1.9–6.1) |
|  | *P* value |  |  | **0.044** |  | 0.522 |  | 0.070 |
| Age group in years | 4–6 | 94 | 23 | 24.5 (16.9–34.1) | 22 | 23.4 (16.0–32.9) | 2 | 2.1 (0.6–7.4) |
|  | 7–10 | 386 | 93 | 24.1 (20.1–28.6) | 101 | 26.2 (22.0–30.8) | 20 | 5.2 (3.4–7.9) |
|  | 11–14 | 158 | 44 | 27.8 (21.5–35.3) | 36 | 22.8 (16.9–29.9) | 10 | 6.3 (3.5–11.3) |
|  | *P* value |  |  | 0.650 |  | 0.663 |  | 0.326 |
| Site | Bafia | 99 | 3 | 3.0 (1.0–8.5) | 4 | 4.0 (1.6–9.9) | 0 | 0 (0.0–0.0) |
|  | Ikata | 148 | 5 | 3.4 (1.5–7.7) | 33 | 22.3 (16.3–29.7) | 0 | 0 (0.0–2.5) |
|  | Likoko | 391 | 152 | 38.9 (34.2–43.8) | 122 | 31.2 (26.8–36.0) | 32 | 8.2 (5.9–11.3) |
|  | *P* value |  |  | **< 0.001** |  | **< 0.001** |  | **< 0.001** |
| Use of potable water (tap) source | Yes | 142 | 7 | 4.9 (2.4–9.8) | 7 | 4.9 (2.4–9.8) | 2 | 1.4 (0.4–5.0) |
|  | No | 496 | 153 | 30.8 (26.9–35.1) | 152 | 30.6 (26.8–34.8) | 30 | 6.1 (4.3–8.5) |
|  | *P* value |  |  | **< 0.001** |  | **< 0.001** |  | **0.026** |
| Bathing site | Home | 140 | 16 | 11.4 (7.2–17.8) | 22 | 15.7 (10.6–22.7) | 2 | 1.4 (0.4–5.1) |
|  | Stream | 479 | 143 | 29.9 (25.9–34.1) | 136 | 28.4 (24.5–32.6) | 30 | 6.3 (4.4–8.8) |
|  | Both | 19 | 1 | 5.3 (0.9–24.6) | 1 | 5.3 (0.9–24.6) | 0 | 0 (0.0–16.8) |
|  | *P* value |  |  | **< 0.001** |  | **< 0.001** |  | **0.042** |
| Distance to water source | Far | 308 | 79 | 25.6 (21.1–30.8) | 83 | 26.9 (22.3–32.2) | 20 | 6.5 (4.2–9.8) |
|  | Near | 330 | 81 | 24.5 (20.2–29.5) | 76 | 23.0 (18.8–27.9) | 12 | 3.6 (2.1–6.7) |
|  | Test |  |  | 0.748 |  | 0.253 |  | 0.098 |
| Nature of house | Plank | 578 | 147 | 25.4 (22.1–29.1) | 147 | 25.4 (22.1–29.1) | 28 | 4.8 (3.4–6.9) |
|  | Block | 60 | 13 | 21.7 (13.1–33.6) | 12 | 20.0 (11.8–31.8) | 4 | 6.7 (2.6–15.9) |
|  | Test |  |  | 0.522 |  | 0.354 |  | 0.538 |
| Open defecation behaviour | Yes | 557 | 151 | 27.1 (23.6–31.0) | 147 | 26.4 (22.9–30.2) | 29 | 5.2 (3.7–7.4) |
|  | No | 81 | 9 | 11.1 (6.0–19.8) | 12 | 14.8 (8.7–24.1) | 3 | 3.7 (1.3–10.3) |
|  | *P* value |  |  | **0.002** |  | **0.024** |  | 0.563 |
| BMI | Normal | 535 | 134 | 25.1 (21.6–28.9) | 140 | 26.2 (22.6–30.1) | 27 | 5.1 (3.5–7.2) |
|  | Thin | 23 | 7 | 30.4 (15.6–50.9) | 4 | 17.4 (7.0–37.1) | 2 | 8.7 (2.4–26.8) |
|  | Obese | 75 | 16 | 21.3 (13.6–31.9) | 13 | 17.3 (10.4–27.4) | 2 | 2.7 (0.7–9.2) |
|  | *P* value |  |  | 0.640 |  | 0.178 |  | 0.463 |

BMI: body mass index; STH: soil-transmitted helminths.
